# Measuring Professionalism in Medicine and Nursing: Results of a European Survey

**DOI:** 10.1371/journal.pone.0097069

**Published:** 2014-05-21

**Authors:** Kiki M. J. M. H. Lombarts, Thomas Plochg, Caroline A. Thompson, Onyebuchi A. Arah, on behalf of the DUQuE Project Consortium

**Affiliations:** 1 Professional Performance Research Group, Center for Evidence-Based Education, Academic Medical Center, University of Amsterdam, Amsterdam, The Netherlands; 2 Department of Public Health, Academic Medical Center, University of Amsterdam, Amsterdam, The Netherlands; 3 Department of Epidemiology, Fielding School of Public Health, University of California Los Angeles, Los Angeles, California, United States of America; 4 Palo Alto Medical Foundation Research Institute, Palo Alto, California, United States of America; 5 UCLA Center for Health Policy Research, Los Angeles, California, United States of America; S.G.Battista Hospital, Italy

## Abstract

**Background:**

Leveraging professionalism has been put forward as a strategy to drive improvement of patient care. We investigate professionalism as a factor influencing the uptake of quality improvement activities by physicians and nurses working in European hospitals.

**Objective:**

To (i) investigate the reliability and validity of data yielded by using the self-developed professionalism measurement tool for physicians and nurses, (ii) describe their levels of professionalism displayed, and (iii) quantify the extent to which professional attitudes would predict professional behaviors.

**Methods and Materials:**

We designed and deployed survey instruments amongst 5920 physicians and nurses working in European hospitals. This was conducted under the cross-sectional multilevel study “Deepening Our Understanding of Quality Improvement in Europe” (DUQuE). We used psychometric and generalized linear mixed modelling techniques to address the aforementioned objectives.

**Results:**

In all, 2067 (response rate 69.8%) physicians and 2805 nurses (94.8%) representing 74 hospitals in 7 European countries participated. The professionalism instrument revealed five subscales of professional attitude and one scale for professional behaviour with moderate to high internal consistency and reliability. Physicians and nurses display equally high professional attitude sum scores (11.8 and 11.9 respectively out of 16) but seem to have different perceptions towards separate professionalism aspects. Lastly, professionals displaying higher levels of professional attitudes were more involved in quality improvement actions (physicians: b = 0.019, *P*<0.0001; nurses: b = 0.016, *P*<0.0001) and more inclined to report colleagues’ underperformance (physicians – odds ratio (OR) 1.12, 95% CI 1.01–1.24; nurses – OR 1.11, 95% CI 1.01–1.23) or medical errors (physicians – OR 1.14, 95% CI 1.01–1.23; nurses – OR 1.43, 95% CI 1.22–1.67). Involvement in QI actions was found to increase the odds of reporting incompetence or medical errors.

**Conclusion:**

A tool that reliably and validly measures European physicians’ and nurses’ commitment to professionalism is now available. Collectively leveraging professionalism as a quality improvement strategy may be beneficial to patient care quality.

## Introduction

The quality of patient care is highly dependent on the performance of physicians and nurses. Although the commitment to the quality of patient care is firmly grounded in the ethical bases of both the medical and nursing professions, ideas about how this commitment should translate in assuring and improving patient care quality have changed over the past decades due to i.e. the explosion of medical knowledge, increased accountability and cost-containment demands and the establishment of the science of quality improvement research. Modern views on professionals’ responsibilities – widely discussed in the context of changing professionalism – have been laid out in some leading documents for physicians and nurses: the Physician charter [1] and the Code of Ethics for Nurses. [2] Both documents stress that today’s professionals need to consider not only what is right and good for individual patients, but to care for all patients and thus for society as a whole. [1], [3], [4] For this purpose, professionals are called to commit to the redefined fundamentals and principles of professionalism, entailing commitments to professional competence, to honesty with patients and to improving the quality of care. The latter needs to reflect the progress that has been made in the discipline of quality improvement [3], including the engagement of physicians and nurses in systematic (organizational) quality improvement activities [5].

More than once, collectively leveraging professionalism has been put forward as the approach to improve the health system [5]–[7]. Given this claim, we were interested in understanding professionals’ attitudes towards the (re)new(ed) professional responsibilities and the related professional behaviors in terms of physicians’ and nurses’ participation in quality improvement activities and them acting upon personal observations of below standard care.

Several reviews on the assessment of professionalism have shown that the measurement of professionalism is problematic [8]–[13]. The reviews identified many different methods for assessing professionalism, a lack of consensus on the definition of professionalism, changing views of professionalism over time and the limited reporting of validity and reliability issues [10], [12]. In addition, most assessment instruments are nationally developed and employed and a validated tool for use at a European level is not yet available.

Against this background, we aimed to develop a multi-faceted tool to capture professional attitudes and behaviors of both physicians and nurses across Europe to empirically investigate their levels of professionalism. For this purpose, we adopted the broad concept of professionalism as it was operationalized in the high impact frameworks mentioned before.

This study was conducted in the context of the DUQuE (Deepening our Understanding of Quality Improvement in Europe) project, which focuses on quality management in European hospitals. Professionalism in the context of the DUQuE project was defined as ‘a set of attitudes and behaviors of professional staff (physicians and registered nurses) that is distinct but related to organisational culture and has implications for individual motivations, teamwork and professional-patient interaction”. We build upon the notion that professional behaviours are expressions of professional attitudes. Therefore, we consider professional attitude to be a predictor of displaying professional behaviours. We will test this hypothesis in this study. More specifically, this study’s research question is threefold: (i) to investigate the reliability and validity of data yielded by using the professionalism measurement tool for physicians and nurses, (ii) to describe levels of professionalism as displayed by physicians and nurses, and (iii) to quantify the extent to which professional attitudes would predict professional behaviors.

## Methods/Design

### Ethics Statement

DUQuE fulfils all the requirements for research projects in the 7th framework of EU DG Research [16]. Ethical approval was obtained by the project coordinator at the Bioethics Committee of the Health Department of the Government of Catalonia (Spain). Each country complied with the confidentiality issues according with national legislation or standards of practice available in each country. All data was anonymous and codes were used for hospitals and countries.

### DUQuE

This study is part of the Deepening our Understanding of Quality improvement in Europe (DUQuE) research project. As suggested by its name, DUQuE builds on the results of its predecessor, the MARQUiS (Methods of Assessing Response to Quality Improvement Strategies) project, which demonstrated substantial variability in the development of hospitals’ quality improvement systems both within and between countries [14], [15]. The overall research objective of the DUQuE project is to study the relationship of quality improvement systems and culture, professionals’ involvement, and patient empowerment with the quality of hospital care in relation to four conditions: acute myocardial infarction (AMI), deliveries, hip fracture and stroke [16]. To address these objectives, the DUQuE project team has conceptualized, adapted and operationalized several measurement tools assumingly relevant to the quality of care in delivered in European hospitals [16]. One of them is the professionalism measurement tool. Although not included in the original DUQuE research proposal, it was decided after ample discussions in the research team, that on theoretical grounds the concept of professional involvement could best be replaced by the two separate phenomena of *professionalism* and *professional involvement*. The latter concept, dealing with physicians and nurses as clinical managers, has been explored in a separate study [17] while this paper focuses on professionalism. The professionalism construct has its place in the overall DUQuE analysis plan as a factor influencing the uptake of quality improvement activities by hospitals (departments) and providing high quality patient care. This will be explored in this and future studies.

### Professionalism Instrument

**Figure 1:**
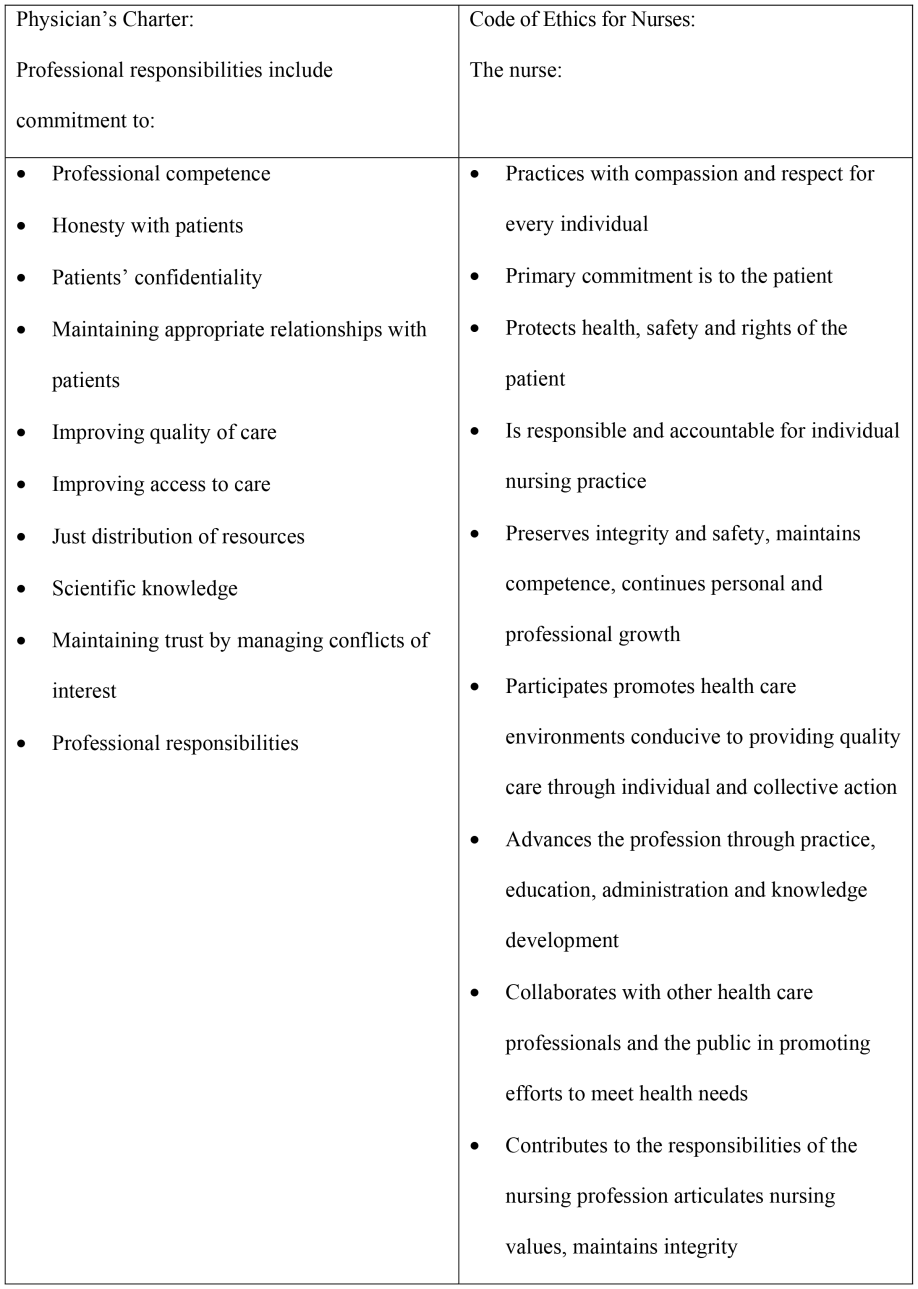
Summary of professional values as defined by the Physician’s Charter (1) and the Code of Ethics for Nurses (2).

In this study, we focus on measuring the level of professionalism of both physicians and (registered) nurses. We developed the professionalism questionnaire building on aspects of professionalism as put forward in two leading documents for physicians and nurses respectively, the Physician’s charter on professionalism [1], defining three principles and ten commitments to professionalism which have achieved worldwide consensus amongst the medical community, and the Code of Ethics for Nurses [2] concerning the domain of professional nursing actions, the quality of professional care, patient safety, and norms of the profession [18]. See Figure 1.

Since previous studies reported on professionalism measures for physicians or nurses only, we developed a combined tool selecting constructs and items that had been used and validated before [18]–[20]. In particular, we compared and selected from the themes and questions used by Campbell et al [21] to measure physicians’ conformance with professional norms, based on the Physicians’ Charter, and the items in the Nurses Professional Values Scale, based on the Nurses Code of Ethics, as developed and validated by Weis et al [18]. To include the important theme of working collaboratively with other professionals in continuously improving quality of patient care, we used a set of validated items on inter-professional physician-nurse collaboration as validated by Ward et al [20]. Given the quality improvement context of this study (DUQuE) and questionnaire length constraints, in the combined tool, we chose to cover the themes from the professionalism frameworks most relevant to quality improvement.

In this study, displaying attitudes or behaviors as defined in these leading documents would qualify as a high level of professionalism. We designed the professionalism scale to encompass both professional attitudes and behaviors. The attitude scale included 4 subscales and multiple items: improving quality of care (4 items), maintaining professional competence (3 items), fulfilling professional responsibilities (4 items) and interprofessional collaboration (7 items). Professional behaviors consisted of 6 primary questions and 2 feeder questions. Attitude questions were answered on a 5-point Likert scale (1 =  strongly disagree, 2 =  somewhat disagree, 3 =  neutral, 4 =  somewhat agree and 5 =  strongly agree). The professional behaviour items all required a yes or no answer. In addition, we collected some specific demographic data. For all we collected data on profession (attending physician, resident in training, registered nurse), age, sex, number of years since completion of professional training and membership of a (national) professional society.

### Study Setting and Participants

In total 2960 physicians and 2960 nurses representing 74 hospitals in Czech Republic, France, Germany, Poland, Portugal, Spain and Turkey were invited to participate in the questionnaire study. All physicians and nurses approached practiced in one of the four hospital departments covered by this project: cardiology, obstetrics, neurology or orthopedics. Questionnaires were translated from English into 7 languages using standard scientific forward-backward translation procedures. [22] The questionnaires were made available electronically via a dedicated web portal. The data collection took place in the period May 2011 to March 2012.

### Data Analysis

After describing the study sample using appropriate statistics, we used psychometric and multivariable regression techniques to investigate the structure, reliability, validity and the interrelationships between the domains of the construct “professionalism” among clinical pathway physicians and nurses. We investigated the factor structure of the questionnaire for each of the four clinical pathways and for nurses and physicians separately using split file principal components analysis with varimax rotation. We retained factors or subscales with an Eigenvalue of at least one and three or more item loadings [23], [24]. Individual items were assigned to the subscale on which they had the highest factor loading, with a minimum acceptable loading being 0.30. If an item loaded equally well on two subscales, subject matter knowledge was used to choose the assigned subscale. We examined internal consistency reliability using Cronbach’s alpha, with an alpha of at least 0.70 taken as acceptable [25], [26]. We then further examined the homogeneity of each subscale using item-total correlation (corrected for item overlap), taking a value above 0.40 as acceptable. We also assessed the degree of redundancy between the subscales using Pearson’s correlation coefficient, such that a correlation coefficient of less than 0.70 was seen as evidence of non-redundant subscales [26], [27]. We computed the mean scores for scales, subscale and items among physicians and nurses separately to gain insights into their distributions. For constructs that comprised more than one subscale, namely professional attitudes, the subscales were summed to an index measure. Using convenient literature-based hypothesis testing [23], [26], [27], we further investigated the validity of the professional attitudes by examining the relationship between it and two assumed predictors of professionalism: membership in a professional society and years since completion of professional training, for physicians and nurses separately. We used generalized linear mixed models with identity link, accounting for clustering within hospitals and adjusted for country effects, hospital level number of beds, teaching status, public versus private ownership, and participant’s age. To investigate whether professional attitudes could predict professional behaviours, we fit multivariable adjusted generalized linear mixed models with logit link (reporting odds ratios and their 95% confidence intervals). All analyses were done in SAS version 9.3 (SAS Inc., Cary, NC; 2012).

## Results

### Study Participants

In total, 4872 professionals (82.3% response rate) participated in this study, including 2067 physicians (69.8%; of which 84% were attendings and 16% residents in training) and 2805 nurses (94.8%), representing 74 hospitals in the seven DUQuE countries. Responding professionals were relatively equally divided over the 4 care pathways or departments covered in this study. Physicians (attendings and residents) were mostly male (59.2%), and nurses mostly female (88.3%). Mean age of all professionals was 39.2 years, and they had worked a mean of 14.4 years since completion of their training. Over 81% of the physicians, but only 44% of the nurses, were members of relevant professional societies. Details on the study sample are reported in tables 1 and 2.

**Table 1:** Characteristics of hospitals participating in study.

Characteristic	N	%
All Hospitals	74	(100)
Czech Republic	12	(16.2)
France	11	(14.8)
Germany	4	(5.4)
Poland	12	(16.2)
Portugal	11	(14.8)
Spain	12	(16.2)
Turkey	12	(16.2)
Teaching Hospitals	33	(44.5)
Public Hospitals	59	(79.7)
Approximate number of beds in hospital		
* <200*	7	(9.4)
* 200–500*	22	(29.7)
* 501–1000*	31	(41.8)
* >1000*	14	(18.9)

**Table 2:** Characteristics of professionalism survey respondents (grouping attending physicians and residents together)^1^.

Characteristics	All Respondents		Physicians^2^		Nurses	
Total number of respondents, N (%)	4872	(100)	2067	(42.4)	2805	(57.5)
Condition pathway, N (%)						
Acute Myocardial Infarction	1238	(25.4)	534	(25.8)	704	(25.0)
Deliveries	1166	(23.9)	528	(25.5)	638	(22.7)
Hip Fracture	1198	(24.5)	490	(23.7)	708	(25.2)
Stroke	1270	(26.0)	515	(24.9)	755	(26.9)
Gender, N (%)						
Male	1524	(31.2)	1223	(59.1)	301	(10.7)
Female	3309	(67.9)	830	(40.1)	2479	(88.3)
Gender missing	39	(0.8)	14	(0.6)	25	(0.8)
Age (years), Mean (SD)	39.2	(9.7)	40.9	(10.0)	38.0	(9.2)
Age missing, N (%)	72	(0.0)	36	(0.0)	36	(0.0)
Number of years since completion of professional training, Mean (SD)	14.4	(10.1)	13.1	(10.3)	15.3	(9.8)
0–5 years, N (%)	1158	(23.7)	598	(28.9)	560	(19.9)
6–10 years, N (%)	803	(16.4)	367	(17.7)	436	(15.5)
11–20 years, N (%)	1417	(29.0)	525	(25.3)	892	(31.8)
21+ years, N (%)	1318	(27.0)	476	(23.0)	842	(30.0)
Years since training missing, N (%)	176	(0.0)	101	(4.8)	75	(2.6)
Member of professional society, N (%)						
Yes	2922	(59.9)	1681	(81.3)	1241	(44.2)
No	1883	(38.6)	364	(17.6)	1519	(54.1)
Professional society missing	67	(1.3)	22	(1.0)	45	(1.6)

1 Excluding professionals who are missing responses for >2 out of 5 professional attitudes subscales.

2 Includes attending physicians and residents-in-training.

### Structure, Reliability and Validity of the Professionalism Instrument

Table 3 provides an overview of the professionalism scale factor loadings, Cronbach’s alpha, and corrected item-total correlations for both physicians and nurses separately. Principal components analysis resulted in the same factor structure for physicians and nurses, revealing 5 subscales of professional attitude, namely ‘improving quality of care’ (items Q1–Q4), ‘maintaining professional competence’ (items PC1–PC3), ‘fulfilling professional responsibilities’ (items PR1–PR4), ‘inter-professional education and collaboration’ (items IC1–IC5) and ‘physician authority’ (items PA1–PA2). For professional behaviours, we found one factor named ‘professional quality improvement actions’ (items QA1–QA3). In the physicians’ scale, 4 of the 6 scales achieved overall moderate to good factor loadings (0.506–0.798). The subscales ‘maintaining professional competence’ and ‘professional quality improvement actions’ each contained one item with a lower factor loading (PC3∶0.349 and QA3∶0.344 respectively).

**Table 3:** Item and scale characteristics, internal consistency, reliability and item-total correlations, by profession.

Item nr	Scale and items	Factor loadings on primary scale		Internal consistency reliability: Cronbach’s α		Corrected item-total correlations	
		Physicians	Nurses	Physicians	Nurses	Physicians	Nurses
	***Improving Quality of Care (N = 2055/2769)*** 1			0.825	0.813		
Q1	Physicians and nurses should be willing to work on quality improvement initiatives.	0.766	0.717			0.665	0.630
Q2	Physicians and nurses should initiate actions to improve daily practice.	0.798	0.751			0.701	0.664
Q3	Physicians and nurses should engage in ongoing self-evaluation.	0.748	0.749			0.694	0.682
Q4	Physicians and nurses should participate in peer evaluations of the quality of care provided by colleagues.	0.604	0.629			0.541	0.555
	***Maintaining Professional Competence (N = 2056/2787)*** 1			0.668	0.664		
PC1	Physicians and nurses should maintain competency in their area of practice.	0.738	0.681			0.560	0.530
PC2	Physicians and nurses should seek additional education to update knowledge and skills.	0.765	0.710			0.617	0.577
PC3	Physicians and nurses should undergo recertification/revalidation examinations periodically throughout their career.	0.349	0.411			0.294	0.337
	***Fulfilling Professional Responsibilities (N = 2051/2780)*** 1			0.765	0.806		
PR1	Physicians and nurses should disclose all significant medical errors to affected patients and/or guardians.	0.597	0.662			0.518	0.583
PR2	Physicians and nurses should report all significant medical errors they observe to hospital, clinic, or other relevant authorities.	0.758	0.783			0.650	0.702
PR3	Physicians and nurses should report all instances of significantly impaired or incompetent colleagues to hospital, clinic, or other relevant authorities.	0.735	0.724			0.635	0.647
PR4	Physicians and nurses should confront practitioners with questionable or inappropriate practice.	0.526	0.623			0.460	0.559
	***Interprofessional Collaboration - Shared education and collaboration (N = 2039/2752)*** 1			0.780	0.771		
IC1	Physicians should be educated to establish collaborative relationships with nurses.	0.738	0.765			0.609	0.628
IC2	Interprofessional relationships between physicians and nurses should be included in their educational programs.	0.745	0.764			0.615	0.634
IC3	Nurses should also have responsibility for monitoring the effects of medical treatment.	0.600	0.495			0.543	0.441
IC4	Nurses should clarify a physician’s order when they feel that it might have the potential for detrimental effects on the patient.	0.539	0.576			0.487	0.528
IC5	A nurse should be viewed as a collaborator and colleague with a physician rather than his/her assistant.	0.574	0.551			0.521	0.487
	***Interprofessional Collaboration - Physician Authority (N = 2045/2763)*** 1			0.543	0.721		
PA1	Doctors should be the dominant authority in all healthcare matters.	0.506	0.664			0.373	0.563
PA2	The primary function of the nurse is to carry out physician’s orders.	0.506	0.664			0.373	0.563
	**Professional Behaviours**						
	***Professional Quality Improvement Actions (N = 2026/2743)*** 2			0.505	0.492		
QA1	In the last 3 years, have you participated in a formal error reduction initiative in your hospital?	0.506	0.477			0.353	0.327
QA2	In the last 3 years, have you reviewed medical/nursing records for quality improvement reasons?	0.523	0.508			0.375	0.364
QA3	In the last 3 years, have you undergone competency assessment by a professional society or other authority (i.e., insurance company)?	0.344	0.353			0.239	0.242

1 Sample size (for physicians/nurses), excludes respondents who are missing responses for >2 out of 5 professional attitudes subscales.

2 Sample size for physicians/nurses.

The nurses’ scale overall showed slightly better factor loadings for 4 of the 6 constructs (0.623–0.783). The items PC3 and QA3 also performed less in this scale with factor loadings of 0.411 and 0.353 respectively. In addition, lower factor loadings were achieved for items IC3 (0.495) and QA1 (0.477). In both professionalism instruments Cronbach’s alphas were good for the constructs ‘improving quality of care’ (0.825 for physicians and 0.813 for nurses), ‘fulfilling professional responsibilities’ (0.765 and 0.806) and ‘inter-professional education and collaboration’ (0.78 and 0.771). The ‘physician authority’ scale was good for nurses (0.721) but weak for physicians (0.543). For both instruments ‘maintaining professional competence’ achieved a borderline acceptable Cronbach’s alpha (0.668 for physicians and 0.664 for nurses) and the Cronbach’s alpha’s was poor for the professional behaviours scale (0.505 for physicians and 0.492 for nurses). In both, the professionalism scales the item-total correlations were all well above 0.40 for all items within their composite-scale, with the exception of item PC3 in the ‘maintaining professional competence’ scale (0.294 for physicians and 0.337 for nurses), and the 3 items in the professional behaviors construct. For the physicians’ instrument the item-total correlation for items PA1 and PA2 of the ‘physician authority’ scale was 0.373.

The factor analyses repeated for physicians and nurses per clinical pathway did not reveal new structures. The pathway specific results are listed in Tables S1 and S2.

For the physicians’ instruments the inter-scale correlations ranged from 0.31 between ‘improvement of quality care’ and ‘inter-professional collaboration‘ to 0.60 between ‘improvement of quality care’ and ‘maintaining professional competence’ (Table 4). For the nurses’ instrument, these numbers were comparable. All numbers were below the Pearson’s correlation coefficient threshold of 0.70 and so the attitudinal subscales can be considered non-redundant. For both instruments, the inter-scale correlations between the professional attitudes constructs and professional behaviours were close to zero.

**Table 4:** Inter-scale correlations for physicians and nurses separately.

	Professional Attitudes						Professional Behaviors
	Q	PC	PR	IC	SEC	PhA	QA
**Physicians**							
***Professional Attitudes Index***							
Improving Quality of Care (Q)	1						
Maintaining Professional Competence (PC)	0.60	1					
Fulfilling Professional Responsibilities (PR)	0.38	0.40	1				
Interprofessional Collaboration (IC)	0.31	0.35	0.35	1			
Shared Education and Collaboration (SEC)	0.47	0.43	0.43	0.60	1		
Physician Authority (PhA)	0.02	0.10	0.10	0.79	−0.03	1	
***Professional Behaviors***							
Professional Quality Improvement Actions (QA)	0.05	−0.01	0.06	0.02	0.04	0.00	1
**Nurses**							
***Professional Attitudes Index***							
Improving Quality of Care (Q)	1						
Maintaining Professional Competence (PC)	0.64	1					
Fulfilling Professional Responsibilities (PR)	0.50	0.48	1				
Interprofessional Collaboration Index (IC)	0.21	0.24	0.27	1			
Shared Education and Collaboration (SEC)	0.52	0.52	0.50	0.35	1		
Physician Authority (PhA)	−0.04	−0.01	0.03	0.88	−0.14	1	
***Professional Behaviours***							
Professional Quality Improvement Actions (QA)	0.11	0.10	0.07	−0.02	0.10	−0.07	1

In multivariate mixed linear models, we detected a positive relationship between membership in a national professional society and the summed index score (physicians: b = 0.26, *P* = 0.0319; nurses: b = 0.25, *P* = 0.0128), meaning that being a member of one’s professional society predicts a doctor or nurse to display a more professional attitude, We also found a positive relationship between years since completing professional training and the summed index score (physicians: b = 0.02, *P*<0.0001; nurses: b = 0.01, *P*<0.0001), meaning that being in practice longer, or being older, predicts a more professional attitude (Table 5).

**Table 5:** Validation of professional attitudes index using predictors of professionalism.

Predictor of professionalism	Physicians			Nurses		
	*b*	SE	Pr >|t|	*b*	SE	Pr >|t|
Membership in a national professional society^1^	0.264	0.123	0.0319	0.249	0.100	0.0128
	N = 1933			N = 2580		
Years since completing professional training^2^	0.019	0.004	<.0001	0.008	0.004	0.0444
	N = 1886			N = 2576		

1 Multivariate linear mixed model with random intercept by hospital, adjusted for fixed effects at the country level (country), hospital level (number of beds, teaching status, and ownership) and patient level (age). Coefficient represents increase in professional attitudes index for individuals who are members of a professional society (compared to those who are not).

2 Multivariate linear mixed model with random intercept by hospital, adjusted for fixed effects at the country level (country), and hospital level (number of beds, teaching status, ownership). Coefficient represents increase in professional attitudes index per 1-year increase in number of years since completing their professional training.

### Levels of Professionalism among Physicians and Nurses

Table 6 reports the levels of professionalism of physicians and nurses expressed as attitudinal and behavioural indices and in terms of their levels of agreement with individual items. The professional attitude index scores for physicians and nurses were both high: 11.8 and 11.9 respectively on a scale ranging 0–16. Physicians scored highest on the attitude scale ‘maintaining professional competence’ (4.3 on a 5 point scale) and lowest on the inter-professional collaboration subscale ‘physician authority’ (3.5 out of 5). Nurses scored highest on the attitude scale ‘improving quality of care’ (4.3 out of 5) and also lowest on the subscale ‘physician authority’ (2.5 out of 5). Most of the professionals (strongly) agreed with the items in the attitudinal scales ‘improving quality of care’ (ranging from 62% to 93% for physicians and ranging from 71% to 95% for nurses) and ‘maintaining professional competence’ (55% to 96% (strong) agreement for physicians; 57% to 96% agreement for nurses).

**Table 6:** Scale mean (SD) scores, and item median (IQR) scores for physicians and nurses separately.

Item nr	Scale and items	Mean (SD)/Median (Q1–Q3) Score^1^				Respondents who agree^2%^ (CI)			
		Physicians		Nurses		Physicians		Nurses	
	**Professional Attitudes Score** 3	**11.8**	**(2.0)**	**11.9**	**(2.0)**				
	***Improving Quality of Care***	**4.2**	**(0.7)**	**4.3**	**(0.6)**				
Q1	Physicians and nurses should be willingto work on quality improvement initiatives.	5	(4–5)	5	(4–5)	93	(92–94)	95	(95–96)
Q2	Physicians and nurses should initiate actionsto improve daily practice.	5	(4–5)	5	(4–5)	90	(89–92)	93	(93–94)
Q3	Physicians and nurses should engage inongoing self-evaluation.	4	(4–5)	4	(4–5)	76	(74–78)	82	(80–83)
Q4	Physicians and nurses should participatein peer evaluations of the quality of careprovided by colleagues.	4	(3–5)	4	(3–5)	62	(60–64)	71	(69–72)
	***Maintaining Professional Competence***	**4.3**	**(0.6)**	**4.2**	**(0.6)**				
PC1	Physicians and nurses should maintaincompetency in their area of practice.	5	(4–5)	5	(4–5)	96	(95–97)	95	(94–96)
PC2	Physicians and nurses should seek additionaleducation to update knowledge and skills.	5	(4–5)	5	(4–5)	97	(96–98)	96	(95–97)
PC3	Physicians and nurses should undergorecertification/revalidation examinationsperiodically throughout their career	4	(3–4)	4	(3–4)	55	(53–57)	57	(55–59)
	***Fulfilling Professional Responsibilities***	**3.6**	**(0.8)**	**3.9**	**(0.7)**				
PR1	Physicians and nurses should disclose allsignificant medical errors to affected patientsand/or guardians.	4	(3–4)	4	(3–4)	54	(52–56)	59	(57–61)
PR2	Physicians and nurses should report allsignificant medical errors they observeto hospital, clinic, or other relevant authorities.	4	(3–4)	4	(3–5)	65	(63–67)	74	(72–75)
PR3	Physicians and nurses should report allinstances of significantly impaired orincompetent colleagues to hospital, clinic,or other relevant authorities.	4	(3–4)	4	(3–5)	53	(51–55)	67	(65–69)
PR4	Physicians and nurses should confrontpractitioners with questionable orinappropriate practice.	4	(3–5)	4	(4–5)	74	(72–76)	82	(81–83)
	***Interprofessional Collaboration*** 4	**3.7**	**(0.5)**	**3.5**	**(0.6)**				
	***Shared education and collaboration***	**4.0**	**(0.7)**	**4.4**	**(0.6)**				
IC1	Physicians should be educated toestablish collaborative relationshipswith nurses.	4	(4–5)	5	(4–5)	76	(75–78)	93	(92–94)
IC2	Interprofessional relationships betweenphysicians and nurses should be includedin their educational programs.	4	(3–5)	5	(4–5)	70	(68–72)	91	(90–92)
IC3	Nurses should also have responsibility formonitoring the effects of medical treatment.	4	(3–5)	4	(3–5)	70	(68–72)	70	(69–72)
IC4	Nurses should clarify a physician’s orderwhen they feel that it might have the potentialfor detrimental effects on the patient.	4	(4–5)	5	(4–5)	88	(86–89)	91	(90–92)
IC5	A nurse should be viewed as a collaboratorand colleague with a physician ratherthan his/her assistant.	4	(3.5–5)	5	(4–5)	75	(73–77)	92	(91–93)
	***Physician Authority***	**3.5**	**(0.9)**	**2.5**	**(1.2)**				
PA1	Doctors should be the dominant authorityin all healthcare matters.	4	(4–5)	3	(2–4)	77	(75–79)	37	(36–39)
PA2	The primary function of the nurse is tocarry out physician’s orders.	3	(2–4)	2	(1–3)	37	(35–39)	24	(23–26)
	**Professional Behaviors** 5								
	***Professional Quality Improvement Actions***	**0.4**	**(0.3)**	**0.4**	**(0.3)**				
QA1	In the last 3 years, have you participatedin a formal error reduction initiativein your hospital?^5^	0.4	(0.4)	0.4	(0.4)	40	(38–42)	38	(37–40)
QA2	In the last 3 years, have you reviewedmedical/nursing records for qualityimprovement reasons?^5^	0.3	(0.4)	0.2	(0.4)	54	(52–56)	49	(47–51)
QA3	In the last 3 years, have you undergonecompetency assessment by a professionalsociety or other authority (i.e., insurance company)?^5^	0.3	(0.4)	0.2	(0.4)	27	(25–29)	23	(22–25)
	***Professional Reaction to Colleagues’ Underperformance*** 6								
PRC1	If, in the last 3 years, you had direct personalknowledge of a colleague (physician or nurse)who was impaired or incompetent in your hospital,group or practice, did you report that colleague(physician or nurse) to the hospital, professionalsociety, or other relevant authority?^5^N = 664/714^7^	0.4	(0.4)	0.6	(0.4)	45	(41–49)	57	(54–61)
PRC2	Other than the care of you or your familyreceived, if, in the last 3 years you had directpersonal knowledge of a serious medical errorin your hospital, group or practice, did youreport that error to the hospital, professionalsociety, or other relevant authority?^5^N = 540/460^7^	0.4	(0.4)	0.3	(0.4)	39	(35–43)	30	(26–34)

1 Median (Q1–Q3) provided for individual likert scale items (range 1–5), mean (SD) provided for subscales (range 1–5) and binary type items (range 0 or 1).

2 For likert scale items, percent of respondents who “somewhat agree” or “strongly agree”, for binary type items, percent of respondents answering “yes”.

3 Professional attitudes score = sum (improving quality of care, maintaining professional competence, fulfilling professional responsibility, Interprofessional collaboration) – 4 (ranges from 0–16).

4 Interprofessional collaboration = mean of shared education and collaboration and physician authority.

5 All professional behaviour items are binary (Yes/No) type items.

6 Professional reactions to colleagues’ performance not aggregated as a subscale.

7 Sample size restricted to those (physicians/nurses) who observed the specific type of underperformance in the past 3 years.

### Interrelationships between Professional Attitudes and Professional Behaviours

Using multivariate mixed models, we found positive relationships between professional attitudes and professional behaviours. In table 7 we report that the summed professionalism index was positively associated with the quality improvement actions subscale (physicians: b = 0.019 p<0.0001; nurses: b = 0.016, p<0.0001). That is, nurses and physicians who are more committed to professional attitudes, are also more likely to participate in professional quality improvement actions such as medical/nursing record reviews, or competency assessment.

**Table 7:** Relationship between professional attitudes and quality improvement actions.

Effect	Professional Quality Improvement Actions (Score 0–3)					
	Physicians			Nurses		
	*b*	SE	Pr >|t|	*b*	SE	Pr >|t|
Professional attitudes^1^ (score range: 0–16)	0.019	0.004	<.0001	0.016	0.004	<.0001
	N = 1881			N = 2496		

1 Multivariate linear mixed model with random intercept by hospital, adjusted for fixed effects at the country level (country), hospital level (number of beds, teaching status, and ownership) and patient level (gender and age).

In table 8 we report the association of the summed professionalism index with increased odds of reporting impaired or incompetent colleagues (physicians: OR 1.12, 95% CI 1.01–1.24; nurses: OR 1.11, 95% CI 1.01–1.23) and serious medical errors (physicians: OR 1.14, 95% CI 1.01–1.23; nurses: OR 1.43, 95% CI 1.22–1.67). We also found (as in Table 8) that the subscale of professional quality improvement actions was associated with increased odds of reporting impaired or incompetent colleagues (physicians: OR 1.52, 95% CI 1.26–1.83; nurses: OR 1.58, 95% CI 1.30–1.91) and serious medical errors (physicians: OR 1.63, 95% CI 1.33–2.00; nurses: OR 1.29, 95% CI 1.02–1.64). Table 8, in other words, reports that if a physician or nurse displays a more professional attitude or is more actively participating in quality improvement actions, he or she is more likely to report - to the hospital or relevant authority – known medical errors or impaired or incompetent peers.

**Table 8:** Relationships between professional attitudes/quality improvement actions, and response towards colleagues’ underperformance.

Predictor	Reporting impaired or incompetent colleagues to hospital or relevant authorities		Odds of reporting serious medical error to hospital or relevant authorities	
	Physicians	Nurses	Physicians	Nurses
	OR (95% confidence limits)	OR (95% confidence limits)	OR (95% confidence limits)	OR (95% confidence limits)
Professional attitudes^1^ (score range 0–16)	1.12 (1.01, 1.24)	1.11 (1.01, 1.23)	1.14 (1.02, 1.26)	1.43 (1.22, 1.67)
	N = 620	N = 659	N = 516	N = 426
Professional quality improvement actions^1^^,^ 2 (score 0–3)^3^	1.52 (1.26, 1.83)	1.58 (1.30, 1.91)	1.63 (1.33, 2.00)	1.29 (1.02, 1.64)
	N = 611	N = 650	N = 509	N = 417

1 Multivariate linear mixed model with random intercept by hospital, adjusted for fixed effects at the country level (country), hospital level (number of beds, teaching status, and ownership) and patient level (gender and age).

2 Additionally adjusted for professional attitudes index.

3 Professional quality improvement actions modeled as a sum of the yes/no questions QA1–QA3 (range 0–3). Coefficient corresponds to a 1 unit increase (one additional “Yes” response to the question series).

## Discussion

We developed an instrument for measuring professionalism of physicians and nurses working in European hospitals yielding valid and reliable data. Physicians and nurses display equally high overall levels of professionalism. Professional attitudes were found to predict professional behaviors, in particular professionals’ involvement in quality improvement activities and their inclination to report underperformance or errors to the relevant authorities.

We were able to develop a profession-specific tool for the measurement of physicians’ and nurses’ professionalism. Although we combined measures from various validated instruments developed for different professions, the factor analysis revealed new constructs structured equally for both professional groups. To name the constructs, we used the labels employed by the Physicians’ Charter (i.e. maintaining professional competence, fulfilling professional responsibilities) meaning that the items derived from the existing nursing instrument [18] now have new construct names. Nevertheless, compared to the original instrument, the individual items all showed higher factor loadings. Higher factor loadings were also found for all but one of the inter-professional collaboration items adopted from the attitudinal scale published by Ward et al [19]. Our data revealed the same two collaboration constructs, ‘shared education and collaboration’ and ‘physician authority’, although the latter scale showed lower reliability scores for the physicians-completed questionnaires.

Overall, physicians and nurses report high levels of professionalism, thus endorsing modern principles of professionalism laid out by the medical and nursing professions. However, compared to the results of the Campbell et al [21] survey of professionalism conducted among North American physicians in 2003, we found lower levels of agreement with many of the core statements in the Physicians’ Charter. We could point at the ten-year time gap between Campbell’s study and ours and at the fact that the Charter has been far more intensely discussed in the USA than in any other nation [28]. However, the authors of the Physician’s Charter state that the members of the medical professions all share the role of healer–which has roots extending back to Hippocrates–and, despite the different contexts, should be able to relate and commit to the set of professional responsibilities outlined in the Charter [1]. Our study may suggest the opposite, that is that the big differences in economic, political, legal or organizational contexts in which professionals in Europe and the USA practice, and the wide variations in medical practice may after all have shaped or impacted the professionals’ attitudes and behaviours. This was also put forward by Roland et al in 2011 [7] when they reported significant differences in levels of professionalism between USA and UK doctors. Clearly, the role of various contexts should be researched further.

Perhaps most striking in the reported professionalism scores are the relatively low levels of agreement with statements related to physicians and nurses professional responsibilities, in particular addressing attitudes reporting medical or nursing errors and incompetent colleagues. The low scores may reflect the deeply rooted idea in professional cultures that mistakes are not tolerated [6], an idea that does not fit in with new civic professionalism that calls for transparency and systematic improvement of care at the individual patient and population levels [3]. Other factors that may explain lower levels of professionalism – at least in an American sample of physicians - include gender, age, practice organization and the malpractice environment [19]. As found in our study, low levels of professionalism are in particular worrisome as not all physicians and nurses who do express their agreement with the professional value statement are prepared to act upon it, i.e. deal with incompetence of a colleague or report errors to the relevant authorities. Could they be hindered by moral ambiguity from acting on their expressed professional attitudes? In an American study by DesRoches et al [19], the most frequently cited reasons for physicians not to report impaired or incompetent colleagues was the belief that someone else was taking care of the problem, and the belief that nothing would happen as a result of the report. It is worth investigating if these beliefs also pertain to European professionals or whether there are other reasons why they do not align their professional attitudes and behaviours.

From a quality improvement perspective it is crucial to improve disclosure practice; it is said to enhance patient satisfaction and patients’ trust in physicians’ integrity and could promote higher quality of care [28]. Professionals may question this; the most noteworthy gap is the absence of prospective evidence about whether disclosure indeed improves patient satisfaction [6], [29].

To sustain the public’s trust in the medical and nursing communities, the practice of professionalism should be taken seriously by every professional. Our study suggests that collectively leveraging professionalism among physicians and nurses may be beneficial to the quality of patient care. Higher levels of professional attitudes are reflected in more professional behaviours, in particular among those that more actively participate in quality improvement and act on identified underperformance or medical errors. This should not be labelled, *per se*, as if these professionals are the better performing professionals in terms of clinical outcomes; future work will need to investigate this.

### Strengths and Limitations

We note several strengths and limitations of this study. This study is a first that looked at professionalism among physicians and nurses working in various clinical departments in European countries. In investigating the properties of the instruments, we did not perform separate analyses for each of the seven countries, as this was not permitted under our European (DUQuE) project agreement. Single countries wanting to use the tools to measure professionalism of physicians or nurses may want to validate the tools further in their context.

## Conclusion

We have developed and tested a tool for reliably and validly measuring European physicians’ and nurses’ commitment to professionalism. Professionals’ relative commitment to the practice of disclosing medical errors to patients or reporting underperformance of colleagues raises some concern in terms of their delivering high quality care to patients. Professionals displaying higher levels of professional attitudes also seem to behave more professionally. This suggests that collectively leveraging professionalism, as a quality improvement strategy, might be beneficial to patient care. Future research should investigate the plausible link(s) from professionalism to clinical outcomes.

## Supporting Information

Table S1 **Physicians: item and scale characteristics, internal consistency, reliability and item-total correlations, by pathway.**
(DOCX)

Table S2 **Nurses: item and scale characteristics, internal consistency, reliability and item-total correlations, by pathway.**
(DOCX)
