# Supplementary material for: Measuring Professionalism in Medicine and Nursing: Results of a European Survey
Source: PLoS One. 2014 May 21;9(5):e97069. doi: 10.1371/journal.pone.0097069 (PMC4029578; doi:10.1371/journal.pone.0097069)
Supplement: Table S1 — Physicians: item and scale characteristics, internal consistency, reliability and item-total correlations, by pathway. (DOCX) [file pone.0097069.s001.docx]

**Table S1. Physicians: item and scale characteristics, internal consistency, reliability and item-total correlations, by pathway**

| **Item nr** | **Scale and items** | **Factor loadings on primary scale** | | | | **Internal consistency reliability: Cronbach’s α** | | | | **Corrected item-total correlations** | | | |
| --- | --- | --- | --- | --- | --- | --- | --- | --- | --- | --- | --- | --- | --- |
|  |  | **AMI** | **DEL** | **HIP** | **STR** | **AMI** | **DEL** | **HIP** | **STR** | **AMI** | **DEL** | **HIP** | **STR** |
|  | **Professional Attitudes^1^** |  |  |  |  |  |  |  |  |  |  |  |  |
|  | ***Improving Quality of Care*** |  |  |  |  | 0.826 | 0.801 | 0.837 | 0.832 |  |  |  |  |
| Q1 | Physicians and nurses should be willing to work on quality improvement initiatives. | 0.762 | 0.764 | 0.771 | 0.769 |  |  |  |  | 0.667 | 0.642 | 0.680 | 0.671 |
| Q2 | Physicians and nurses should initiate actions to improve daily practice. | 0.791 | 0.786 | 0.810 | 0.802 |  |  |  |  | 0.695 | 0.673 | 0.723 | 0.707 |
| Q3 | Physicians and nurses should engage in ongoing self-evaluation. | 0.754 | 0.708 | 0.768 | 0.759 |  |  |  |  | 0.698 | 0.656 | 0.715 | 0.705 |
| Q4 | Physicians and nurses should participate in peer evaluations of the quality of care provided by colleagues. | 0.612 | 0.553 | 0.617 | 0.631 |  |  |  |  | 0.547 | 0.490 | 0.559 | 0.564 |
|  | ***Maintaining Professional Competence*** |  |  |  |  | 0.662 | 0.628 | 0.629 | 0.653 |  |  |  |  |
| PC1 | Physicians and nurses should maintain competency in their area of practice. | 0.717 | 0.657 | 0.810 | 0.746 |  |  |  |  | 0.542 | 0.470 | 0.646 | 0.559 |
| PC2 | Physicians and nurses should seek additional education to update knowledge and skills. | 0.749 | 0.719 | 0.813 | 0.765 |  |  |  |  | 0.606 | 0.586 | 0.655 | 0.605 |
| PC3 | Physicians and nurses should undergo recertification/revalidation examinations periodically throughout their career. | 0.361 | 0.355 | 0.363 | 0.313 |  |  |  |  | 0.300 | 0.279 | 0.322 | 0.263 |
|  | ***Fulfilling Professional Responsibilities*** |  |  |  |  | 0.790 | 0.755 | 0.755 | 0.757 |  |  |  |  |
| PR1 | Physicians and nurses should disclose all significant medical errors to affected patients and/or guardians. | 0.650 | 0.599 | 0.608 | 0.535 |  |  |  |  | 0.574 | 0.518 | 0.521 | 0.461 |
| PR2 | Physicians and nurses should report all significant medical errors they observe to hospital, clinic, or other relevant authorities. | 0.818 | 0.715 | 0.739 | 0.754 |  |  |  |  | 0.716 | 0.602 | 0.629 | 0.652 |
| PR3 | Physicians and nurses should report all instances of significantly impaired or incompetent colleagues to hospital, clinic, or other relevant authorities. | 0.759 | 0.713 | 0.723 | 0.744 |  |  |  |  | 0.660 | 0.609 | 0.629 | 0.639 |
| PR4 | Physicians and nurses should confront practitioners with questionable or inappropriate practice. | 0.506 | 0.556 | 0.504 | 0.550 |  |  |  |  | 0.456 | 0.479 | 0.435 | 0.474 |
|  | ***Interprofessional Collaboration - Shared education and collaboration*** |  |  |  |  | 0.791 | 0.759 | 0.805 | 0.761 |  |  |  |  |
| IC1 | Physicians should be educated to establish collaborative relationships with nurses. | 0.710 | 0.744 | 0.722 | 0.773 |  |  |  |  | 0.581 | 0.612 | 0.614 | 0.626 |
| IC2 | Interprofessional relationships between physicians and nurses should be included in their educational programs. | 0.757 | 0.736 | 0.737 | 0.749 |  |  |  |  | 0.636 | 0.599 | 0.625 | 0.593 |
| IC3 | Nurses should also have responsibility for monitoring the effects of medical treatment. | 0.629 | 0.576 | 0.661 | 0.532 |  |  |  |  | 0.568 | 0.521 | 0.599 | 0.482 |
| IC4 | Nurses should clarify a physician’s order when they feel that it might have the potential for detrimental effects on the patient. | 0.557 | 0.471 | 0.600 | 0.527 |  |  |  |  | 0.501 | 0.422 | 0.544 | 0.482 |
| IC5 | A nurse should be viewed as a collaborator and colleague with a physician rather than his/her assistant. | 0.611 | 0.543 | 0.622 | 0.517 |  |  |  |  | 0.559 | 0.483 | 0.567 | 0.467 |
|  | ***Interprofessional Collaboration - Physician Authority*** |  |  |  |  | 0.591 | 0.533 | 0.506 | 0.529 |  |  |  |  |
| PA1 | Doctors shoul d be the dominant authority in all healthcare matters. | 0.545 | 0.497 | 0.476 | 0.494 |  |  |  |  | 0.419 | 0.363 | 0.339 | 0.360 |
| PA2 | The primary function of the nurse is to carry out physician’s orders. | 0.545 | 0.497 | 0.476 | 0.494 |  |  |  |  | 0.419 | 0.363 | 0.339 | 0.360 |
|  |  |  |  |  |  |  |  |  |  |  |  |  |  |
|  | **Professional Behaviours** |  |  |  |  |  |  |  |  |  |  |  |  |
|  | ***Professional Quality Improvement Actions*** |  |  |  |  | 0.451 | 0.541 | 0.509 | 0.517 |  |  |  |  |
| QA1 | In the last 3 years, have you participated in a formal error reduction initiative in your hospital? | 0.480 | 0.564 | 0.503 | 0.486 |  |  |  |  | 0.315 | 0.387 | 0.361 | 0.348 |
| QA2 | In the last 3 years, have you reviewed medical/nursing records for quality improvement reasons? | 0.492 | 0.605 | 0.492 | 0.501 |  |  |  |  | 0.333 | 0.456 | 0.349 | 0.364 |
| QA3 | In the last 3 years, have you undergone competency assessment by a professional society or other authority (i.e., insurance company)? | 0.287 | 0.317 | 0.379 | 0.402 |  |  |  |  | 0.188 | 0.229 | 0.264 | 0.283 |

^1^All professional attitude statistics exclude respondents who are missing responses for >2 out of 5 professional attitudes subscales.
