# Supplementary material for: Measuring Professionalism in Medicine and Nursing: Results of a European Survey
Source: PLoS One. 2014 May 21;9(5):e97069. doi: 10.1371/journal.pone.0097069 (PMC4029578; doi:10.1371/journal.pone.0097069)
Supplement: Table S2 — Nurses: item and scale characteristics, internal consistency, reliability and item-total correlations, by pathway. (DOCX) [file pone.0097069.s002.docx]

**Table S2. Nurses: item and scale characteristics, internal consistency, reliability and item-total correlations, by pathway**

| **Item nr** | **Scale and items** | **Factor loadings on primary scale** | | | | **Internal consistency reliability: Cronbach’s α** | | | | **Corrected item-total correlations** | | | |
| --- | --- | --- | --- | --- | --- | --- | --- | --- | --- | --- | --- | --- | --- |
|  |  | **AMI** | **DEL** | **HIP** | **STR** | **AMI** | **DEL** | **HIP** | **STR** | **AMI** | **DEL** | **HIP** | **STR** |
|  | **Professional Attitudes^1^** |  |  |  |  |  |  |  |  |  |  |  |  |
|  | ***Improving Quality of Care*** |  |  |  |  | 0.798 | 0.807 | 0.842 | 0.806 |  |  |  |  |
| Q1 | Physicians and nurses should be willing to work on quality improvement initiatives. | 0.758 | 0.685 | 0.736 | 0.695 |  |  |  |  | 0.649 | 0.601 | 0.661 | 0.611 |
| Q2 | Physicians and nurses should initiate actions to improve daily practice. | 0.756 | 0.719 | 0.787 | 0.748 |  |  |  |  | 0.639 | 0.640 | 0.713 | 0.663 |
| Q3 | Physicians and nurses should engage in ongoing self-evaluation. | 0.706 | 0.763 | 0.781 | 0.742 |  |  |  |  | 0.647 | 0.688 | 0.719 | 0.672 |
| Q4 | Physicians and nurses should participate in peer evaluations of the quality of care provided by colleagues. | 0.576 | 0.650 | 0.676 | 0.612 |  |  |  |  | 0.507 | 0.562 | 0.610 | 0.541 |
|  | ***Maintaining Professional Competence*** |  |  |  |  | 0.628 | 0.584 | 0.691 | 0.673 |  |  |  |  |
| PC1 | Physicians and nurses should maintain competency in their area of practice. | 0.611 | 0.685 | 0.690 | 0.734 |  |  |  |  | 0.470 | 0.538 | 0.544 | 0.564 |
| PC2 | Physicians and nurses should seek additional education to update knowledge and skills. | 0.641 | 0.699 | 0.736 | 0.759 |  |  |  |  | 0.511 | 0.560 | 0.614 | 0.614 |
| PC3 | Physicians and nurses should undergo recertification/revalidation examinations periodically throughout their career. | 0.430 | 0.406 | 0.448 | 0.365 |  |  |  |  | 0.337 | 0.333 | 0.373 | 0.308 |
|  | ***Fulfilling Professional Responsibilities*** |  |  |  |  | 0.791 | 0.827 | 0.807 | 0.803 |  |  |  |  |
| PR1 | Physicians and nurses should disclose all significant medical errors to affected patients and/or guardians. | 0.654 | 0.678 | 0.669 | 0.652 |  |  |  |  | 0.569 | 0.610 | 0.585 | 0.573 |
| PR2 | Physicians and nurses should report all significant medical errors they observe to hospital, clinic, or other relevant authorities. | 0.780 | 0.785 | 0.785 | 0.786 |  |  |  |  | 0.689 | 0.712 | 0.708 | 0.700 |
| PR3 | Physicians and nurses should report all instances of significantly impaired or incompetent colleagues to hospital, clinic, or other relevant authorities. | 0.703 | 0.752 | 0.690 | 0.754 |  |  |  |  | 0.619 | 0.678 | 0.621 | 0.675 |
| PR4 | Physicians and nurses should confront practitioners with questionable or inappropriate practice. | 0.587 | 0.673 | 0.647 | 0.594 |  |  |  |  | 0.525 | 0.612 | 0.579 | 0.529 |
|  | ***Interprofessional Collaboration - Shared education and collaboration*** |  |  |  |  | 0.754 | 0.783 | 0.755 | 0.790 |  |  |  |  |
| IC1 | Physicians should be educated to establish collaborative relationships with nurses. | 0.788 | 0.727 | 0.781 | 0.762 |  |  |  |  | 0.638 | 0.600 | 0.613 | 0.653 |
| IC2 | Interprofessional relationships between physicians and nurses should be included in their educational programs. | 0.777 | 0.727 | 0.805 | 0.747 |  |  |  |  | 0.630 | 0.611 | 0.651 | 0.642 |
| IC3 | Nurses should also have responsibility for monitoring the effects of medical treatment. | 0.445 | 0.521 | 0.470 | 0.541 |  |  |  |  | 0.401 | 0.456 | 0.419 | 0.481 |
| IC4 | Nurses should clarify a physician’s order when they feel that it might have the potential for detrimental effects on the patient. | 0.511 | 0.641 | 0.546 | 0.616 |  |  |  |  | 0.473 | 0.581 | 0.509 | 0.561 |
| IC5 | A nurse should be viewed as a collaborator and colleague with a physician rather than his/her assistant. | 0.540 | 0.613 | 0.478 | 0.576 |  |  |  |  | 0.468 | 0.550 | 0.425 | 0.511 |
|  | ***Interprofessional Collaboration - Physician Authority*** |  |  |  |  | 0.709 | 0.755 | 0.724 | 0.691 |  |  |  |  |
| PA1 | Doctors should be the dominant authority in all healthcare matters. | 0.652 | 0.698 | 0.667 | 0.635 |  |  |  |  | 0.549 | 0.607 | 0.568 | 0.528 |
| PA2 | The primary function of the nurse is to carry out physician’s orders. | 0.652 | 0.698 | 0.667 | 0.635 |  |  |  |  | 0.549 | 0.607 | 0.568 | 0.528 |
|  |  |  |  |  |  |  |  |  |  |  |  |  |  |
|  | **Professional Behaviours** |  |  |  |  |  |  |  |  |  |  |  |  |
|  | ***Professional Quality Improvement Actions*** |  |  |  |  | 0.465 | 0.475 | 0.506 | 0.517 |  |  |  |  |
| QA1 | In the last 3 years, have you participated in a formal error reduction initiative in your hospital? | 0.461 | 0.465 | 0.507 | 0.485 |  |  |  |  | 0.307 | 0.306 | 0.346 | 0.349 |
| QA2 | In the last 3 years, have you reviewed medical/nursing records for quality improvement reasons? | 0.490 | 0.512 | 0.543 | 0.488 |  |  |  |  | 0.343 | 0.367 | 0.395 | 0.353 |
| QA3 | In the last 3 years, have you undergone competency assessment by a professional society or other authority (i.e., insurance company)? | 0.330 | 0.331 | 0.333 | 0.412 |  |  |  |  | 0.218 | 0.220 | 0.230 | 0.291 |

^1^All professional attitude statistics exclude respondents who are missing responses for >2 out of 5 professional attitudes subscales
